# Comparison of the distribution of lymph node metastases compared to healthy lymph nodes in breast cancer

**DOI:** 10.1186/s13014-021-01964-6

**Published:** 2022-02-05

**Authors:** Kai J. Borm, Lucia Ernst, Julia Voppichler, Markus Oechsner, Mathias Düsberg, Gabriel Buschner, Wolfgang Weber, Stephanie E. Combs, Marciana N. Duma

**Affiliations:** 1 Department of Radiation Oncology, Medical School, Klinikum rechts der Isar, Technical University Munich, Munich, Germany; 2 Department of Nuclear Medicine, Medical School, Klinikum rechts der Isar, Technical University Munich, Munich, Germany; 3 Deutsches Konsortium Für Translationale Krebsforschung (DKTK)-Partner Site Munich, Munich, Germany; 4 Institute of Radiation Medicine, Helmholtzzentrum München, Munich, Germany; 5 Present Address: Department of Radiotherapy and Radiation Oncology, Friedrich Schiller University Hospital, Bachstraße 18, 07743 Jena, Germany

**Keywords:** Breast cancer, Lymph node irradiation, Lymphatic system, Lymph node metastases, Lymph node mapping

## Abstract

**Background:**

Current literature lacks a comparison of lymph node metastases and non-pathological lymph nodes distribution in breast cancer patients. The aim of the current retrospective study was to generate a comprehensive atlas of the lymph node system.

**Methods:**

143 breast cancer patients underwent F-18-FDG-PET/CT (PET/CT) imaging for staging purposes and were diagnosed with regional lymph node metastases. Based on the PET/CT data set a total of 326 lymph node metastases and 1826 non-pathological lymph nodes were detected and contoured manually in the patient collective. Using rigid and deformable registration algorithms all structures were transferred to a template planning CT of a standard patient. Subsequently, a 3D-atlas of the distribution of lymph node metastases and non-pathological lymph nodes were generated and compared to each other.

**Results:**

Both, lymph node metastases and non-pathological lymph nodes, accumulated in certain areas (“hot-spots”) within the lymphatic drainage system. However large differences regarding the distribution patterns were detected: lymph node metastases hot spots occurred in close proximity to the subclavian vein in level I-III, whereas the non-pathological lymph nodes accumulated mostly (within a wider range) in level I. In level II and III lymph node metastases exceeded clearly the areas in which non-pathological lymph nodes occurred.

**Conclusion:**

Lymph node metastases and non-pathological lymph node distribution within the lymph node system differ clearly. Based on our results, an individual adjustment of the CTV in order to include visible lymph nodes in level II and III should be discussed.

## Main points

- lymph node metastases and non-pathological lymph nodes, accumulated in certain areas
- lymph node metastases hot spots occurred in close proximity to the subclavian vein in level I-III, whereas the non-pathological lymph nodes accumulated mostly (within a wider range)
- In level II and III lymph node metastases exceeded clearly the areas in which non-pathological (visible) lymph nodes occurred.

## Introduction

The lymphatic drainage system plays a crucial role in the treatment of breast cancer patients. On the one hand lymph node involvement is an independent risk factor that needs to be taken into consideration for (neoadjuvant) systemic therapy decisions [1–3]. On the other hand, locoregional treatment targeted to the lymphatic drainage system itself has an impact on the disease-free survival and lowers locoregional and distant metastases [4, 5].

In patients with > 3 positive lymph nodes (neo-)adjuvant chemotherapy is usually recommended. In case of 1–3 positive lymph nodes, additional characteristics such as tumor stage and multigene assays need to be considered. Further, if several lymph nodes seem to be involved, surgical axillary lymph node dissection (ALND) is needed [3]. Based on previous reports, the extent of ALND and the number of dissected lymph nodes varies widely between the patients [6, 7]. Several approaches for a better identification and selection of lymph nodes at risk during ALND are currently discussed and investigated [8, 9]. Analyses of the distribution of lymph node metastases (mLN) and comparison with visible non-pathological lymph nodes (npLN) could be helpful for a more targeted approach during axillary surgery for the large majority of patients in which no F-18-FDG-PET/CT exam is available.

In node positive high-risk patients, surgery is often followed by lymph node irradiation. Nonetheless, as adjuvant radiotherapy targets only the microscopic disease, target volume definition is difficult. Large interobserver variations can be seen in clinical practice and even the consensus recommendations that were designed for a more consistent radiotherapy target definition differ in important points [10, 11]. However, a precise delineation of target volumes has a potential impact both on the oncologic outcome as well as on the side effects—i.e. lymphedema. For optimization of the target of the radiation therapy, imaging information about the lymph node system is needed.

Detection and diagnosis of mLN in images is a challenging task. Computed tomography (CT) is regularly used during the staging procedure but provides only limited sensitivity regarding identification of mLN [12]. Thus, additional assessment of the axilla using ultrasonography is needed in clinical practice. Even though F-18-FDG-PET/CT and ultrasonography have a similar accuracy regarding the detection of lymph node metastases [13], PET/CT is not yet a standard modality for breast cancer staging. The information provided in F-18-FDG-PET images can help to distinguish pathological and non-pathological lymph nodes in CT images [12, 14]. Thus, information derived from F-18-FDG-PET/CTs in a large cohort can help to evaluate the current contouring recommendations and improve nodal target definition which is usually based on CT-images only.

Previous studies indicate that mLN accumulate in certain areas within the lymph node drainage system [15]. However, current literature lacks a comprehensive comparison of the distribution of mLN with non-pathological lymph nodes (npLN) in the imaging of breast cancer patients. A comparison based on a large patient collective could enable a more targeted approach during radiotherapy for patients without FDG-PET/CT imaging prior to treatment and help to understand whether visible lymph nodes in the planning CT need should be included in the lymph node CTV.

## Methods and material

The methods were previously described [15]. In summary, from our database, all patients diagnosed with locoregional mLN on F18-FDG-PET/CT (defined as axillary, supraclavicular or internal mammary mLN) by experienced specialists in nuclear medicine and radiology were chosen. 92 Patients with a history of contralateral breast cancer or bilateral lymph node metastases were excluded from the analyses. The remaining 143 patients were divided into 4 groups according to their course of disease (primary vs recurrent breast cancer) and the presence or absence of distant metastasis at the time of the F18-FDG-PET/CT staging (distant metastasis vs. no distant metastasis).

143 patients out of this patient collective were included in the present study. The study was approved by the local ethic committee (xxx) and all patients gave informed consent for the treatment.

### Lymph node contouring and image registration

The diagnostic CT images acquired during F18-FDG-PET/CT scan were imported into the radiotherapy planning software (Eclipse 13.0. Varian Medical Systems. Palo Alto. CA) in order to be delineated. Subsequently all visible axillary, supraclavicular and internal mammary LNs located contralateral to the primary tumor site (i.e. In the contralateral axilla) were contoured. All contoured structures were transferred to the template CT (patient age 50 years; body mass index: 26.6; bust girth: 85 cm; cup size: B) using rigid and nonrigid image registration techniques as previously described [15]. The image registration algorithm was implemented in MATLAB R2017b (The MathWorks. Inc. Natick. MA) and operated in 3 steps. First, a global rigid registration estimated a coarse alignment of the 2 image data sets. In a second step the image data are masked by regions of interest defined by a margin of 5 cm around each LN in each of the regions (axillary, supraclavicular and internal mammary region). For these masked image regions another rigid registration was performed. In a third step, a nonrigid registration inside the regions of interest was performed with the image registration framework plastimatch using the B-Spline algorithm. The algorithm was executed over 6 stages optimizing the mean squared error metric and the calculated deformation vector fields were applied to the contoured structures. All deformed LNs on the standard patient were visually assessed with regard to localization and form.

### Comparison of lymph node distribution

**Fig. 1:**
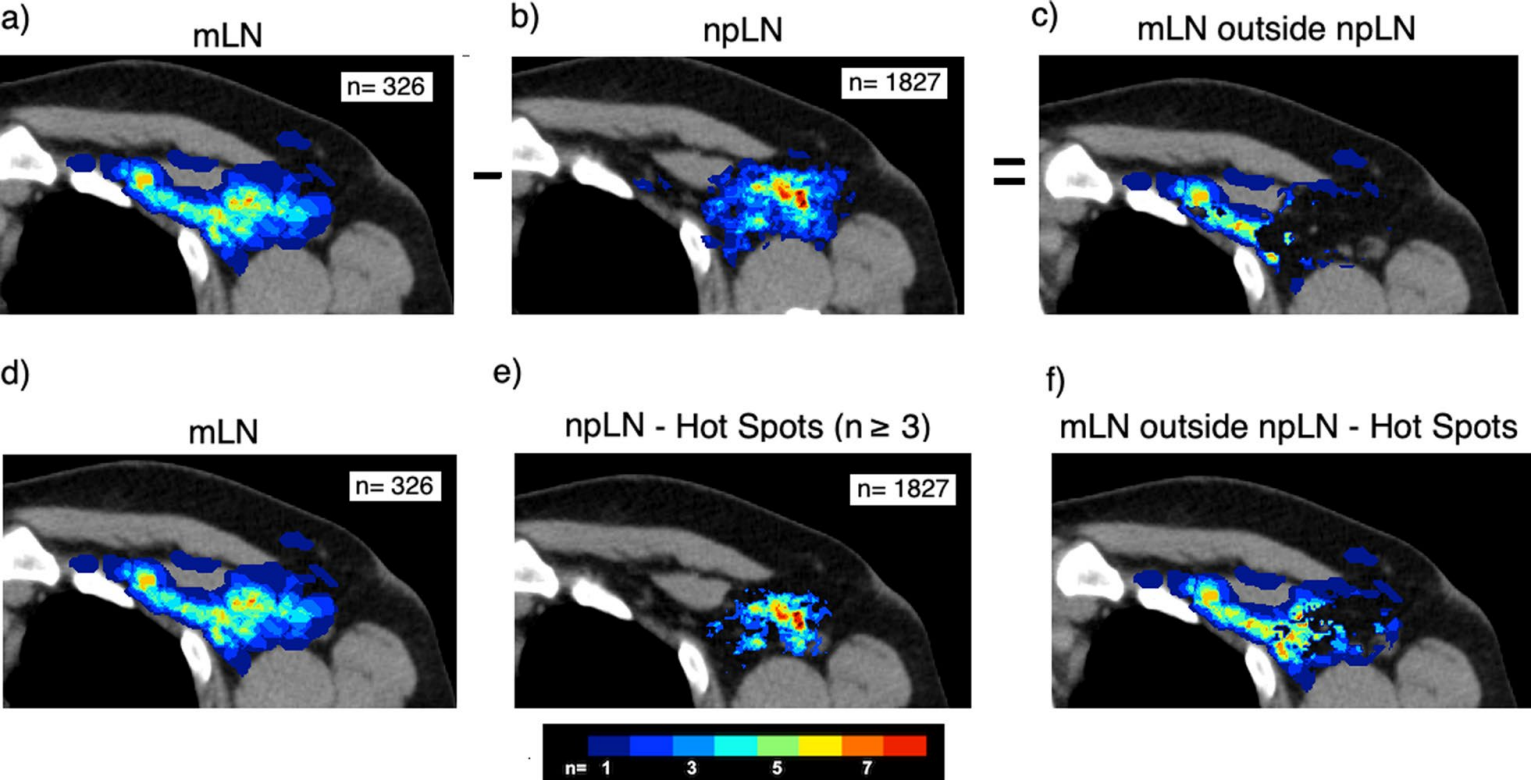
Principle of comparison of LN distribution. Atlas of lymph node metastases (mLN) (**a**, **d**) and non-pathological lymph nodes (npLN) (**b**, **e**). (npLN-Hot Spots (**e**): atlas containing only areas with ≥ 3 npLN). **c**, **f** mLN outside the areas where npLN (**c**) or npLN hot spots (**f**) were observed

The binary masks of all contoured structures were summed up using MATLAB. The resulting number of lymph nodes in each voxel was delineated color-coded. Thus, we created a 3-dimensional atlas representing the physiologic pattern of lymph nodes within the lymphatic drainage system as well as a 3-dimensional atlas of metastatic lymph nodes. The distribution of mLN and contralateral visible npLN were then compared with regard to the distribution in level I-IV and the internal mammary region. In the next step, to detect spatial differences in the distribution of the npLN and mLN we delineated only mLN outside voxels in which npLN occurred. This was implemented by assigning the number 0 in MATLAB in the mLN atlas (“blinding”) to all voxels that were overlapped by npLN. The same procedure was repeated vice versa by blinding all mLN and showing only the surrounding npLN. In the current study we defined “hot spots” as voxel in which ≥ 3 lymph node metastases occurred. For delineation of lymph node “hot spots” we used the same methodology described above but blinded only areas with at least 3 mLN or npLN, respectively. The principle is explained in Fig. 1.

## Results

The primary tumor was located in 83 cases (58.0%) on the left side and in 60 cases (42.0%) on the right side. 47 patients (33.0%) received F-18-FDG-PET/CT staging during primary diagnosis or treatment of breast cancer, the remaining 96 patients (67.0%) had recurrent disease at the time of PET/CT imaging. 70 patients (48.9%) had distant metastases at the time the PET/CT image was acquired. The total number of ipsilateral mLN in the patient collective was 326. The total number of visible npLN contralateral to the primary tumor site was 1827. The mLN were significantly larger compared to npLN: The mean (± SD) volume was 1.9 ± 2.9 cm^3^ (mLN) and 0.2 ± 0.3 cm^3^ (npLN) (*p* < 0.001) and the mean max. diameter 1.4 ± 0.7 cm (mLN) and 0.8 ± 0.4 cm (npLN) (*p* < 0.001). mLN occurred more frequently in level III (11.0% vs. 1.1%) and the internal mammary region (8.3% vs. 2.8%) compared to the npLN. The distribution of mLN and npLN with regard to the lymph node levels is summarized in Table 1.

**Table 1:** Comparison of non-pathologic lymph nodes (npLN) and lymph node metastases (mLN) with regard to the lymph node levels

Lymph node region	npLN		mLN	
	N =	%	N =	%
Axillary level I	1266	69.3%	186	57.1%
Axillary level II	191	10.5%	35	10.7%
Axillary level III	20	1.1%	36	11.0%
Level IV	266	14.6%	31	9.5%
Internal mammary	51	2.8%	27	8.3%
Other	33	1.8%	11	3.4%
Total	1827	100.0%	326	100.0%

**Fig. 2.**
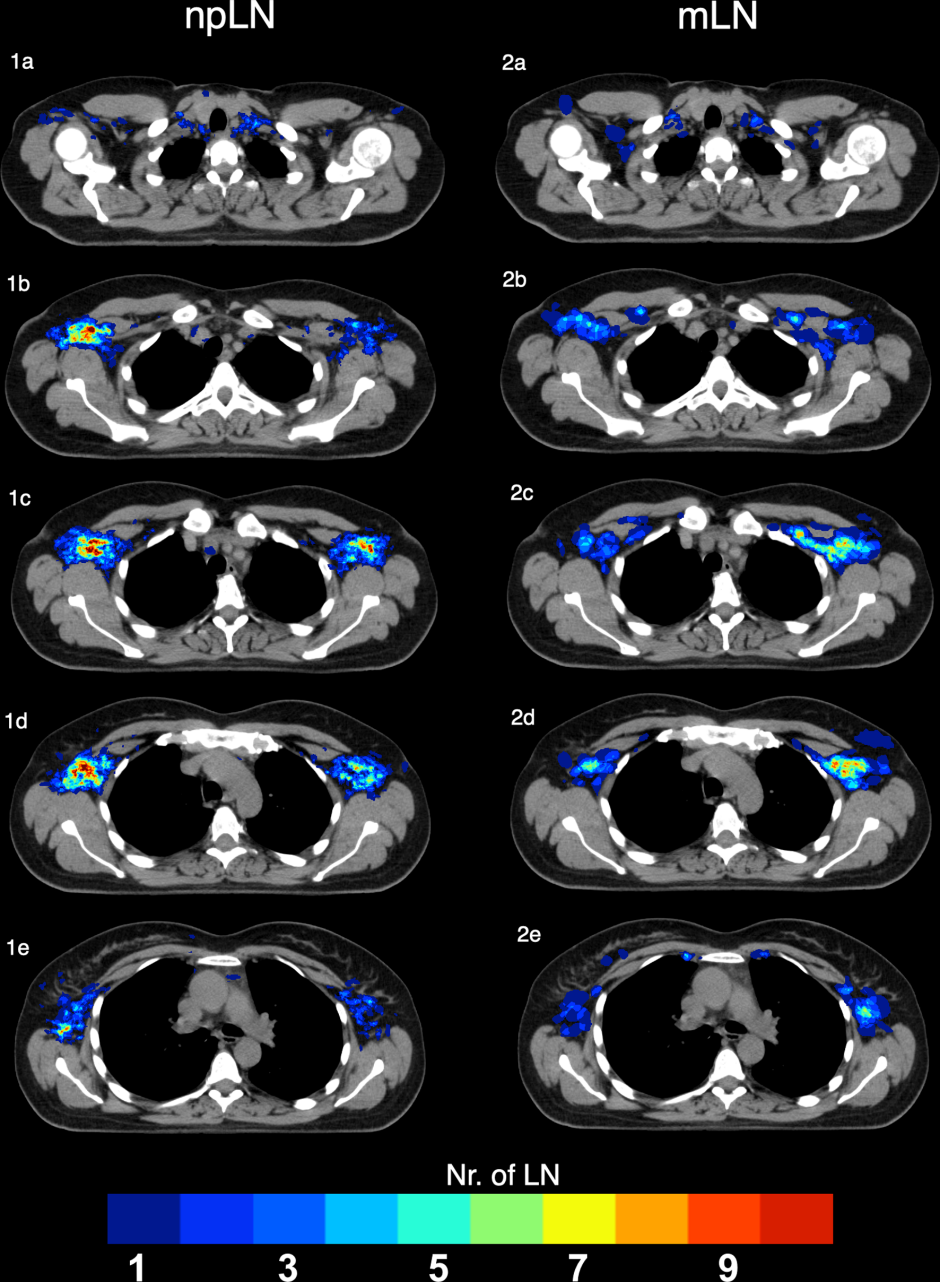
3D-Atlas of Lymph node metastases (mLN) (**1a**–**1e**) and non-pathological lymph nodes contralateral to the primary tumor site (npLN) (**2a**–**2e**)

Figure 2 delineates a npLN and mLN 3D- atlas in CT-slices at different heights. Due to the asymmetric distribution of the primary breast cancer site, more mLN are located on the left side and more npLN on the right side. npLN and mLN accumulated in certain areas: npLN were mostly located in level I dorso-laterally to the pectoralis minor muscle delineated as “hot-spots” in the color-coded atlas (Fig. 21b–1d). The mLN had the highest incidence caudally to the subclavian vein in level I-III (2c–2d).

**Fig. 3.**
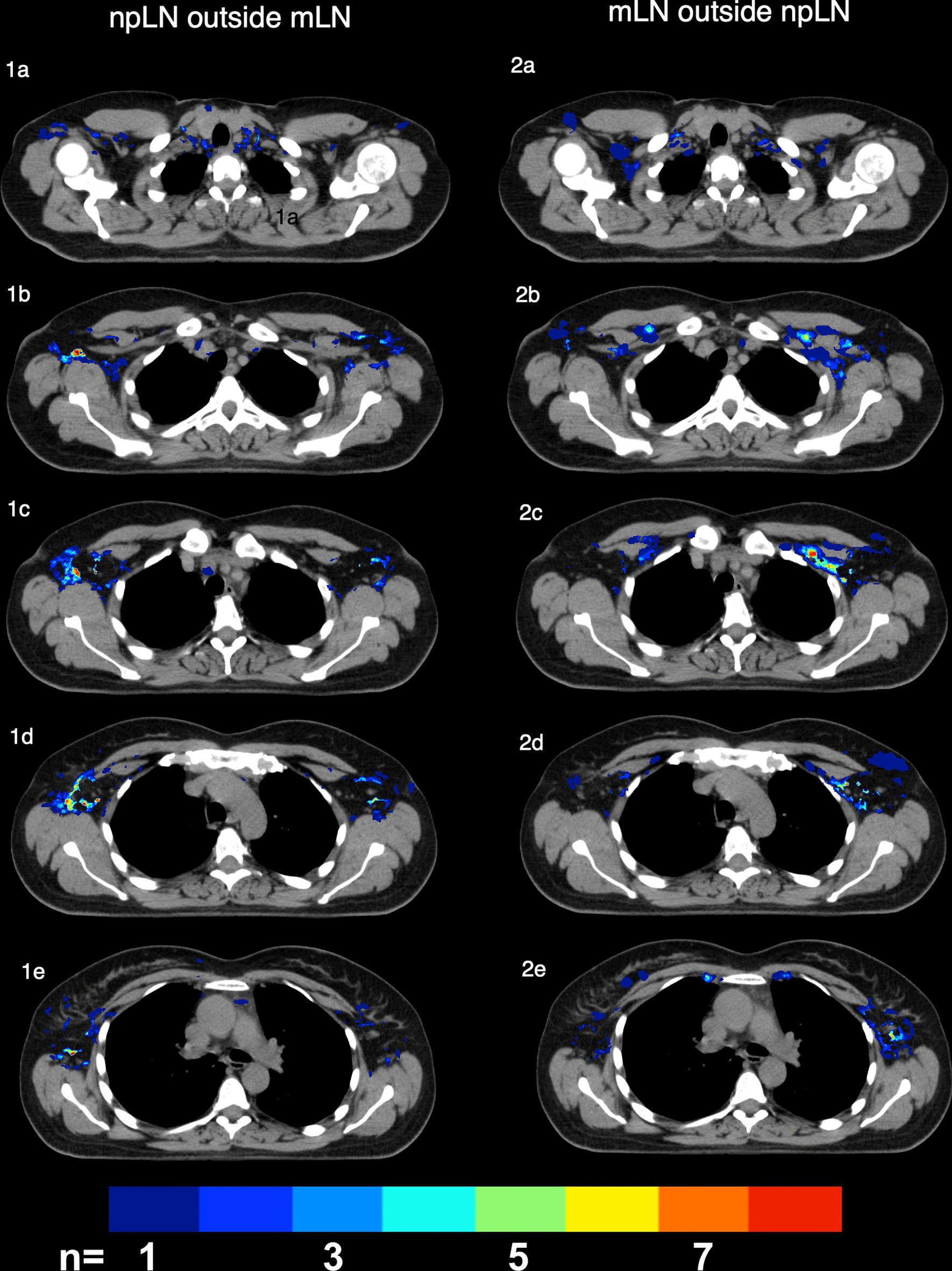
**1a–e** Delineation of lymph node metastases (mLN) outside non-pathological lymph node metastases (npLN) **2a–e** npLN outside mLN by blinding all areas with overlap of npLN and mLN

Figure 3 emphasizes the differences between npLN and mLN by delineating only mLN (or npLN) that occur outside areas were npLN (or mLN, respectively) were observed. In level I, npLN extended more dorsally (around the thoracodorsal vessels) and more laterally (closer to the skin) compared to mLN. mLN on the other hand exceeded npLN areas in close proximity to the chest wall and to the minor pectoral muscle. In Level II and III large number of mLN were located in proximity to the subclavian vein where only few npLN were detected. In the supraclavicular and internal mammary region, the total number of lymph nodes was too small to reliably detect differences between the distribution of npLN and mLN. However, npLN occurred more frequently in close proximity to the trachea and the esophagus, while mLN were seen more often in the peripheral parts of the MS region and closer to the clavicle (Fig. 31a, 2a). Differently from npLN, a small number of mLN were also observed in the rotter space between the minor and major pectoral muscle.

**Fig. 4.**
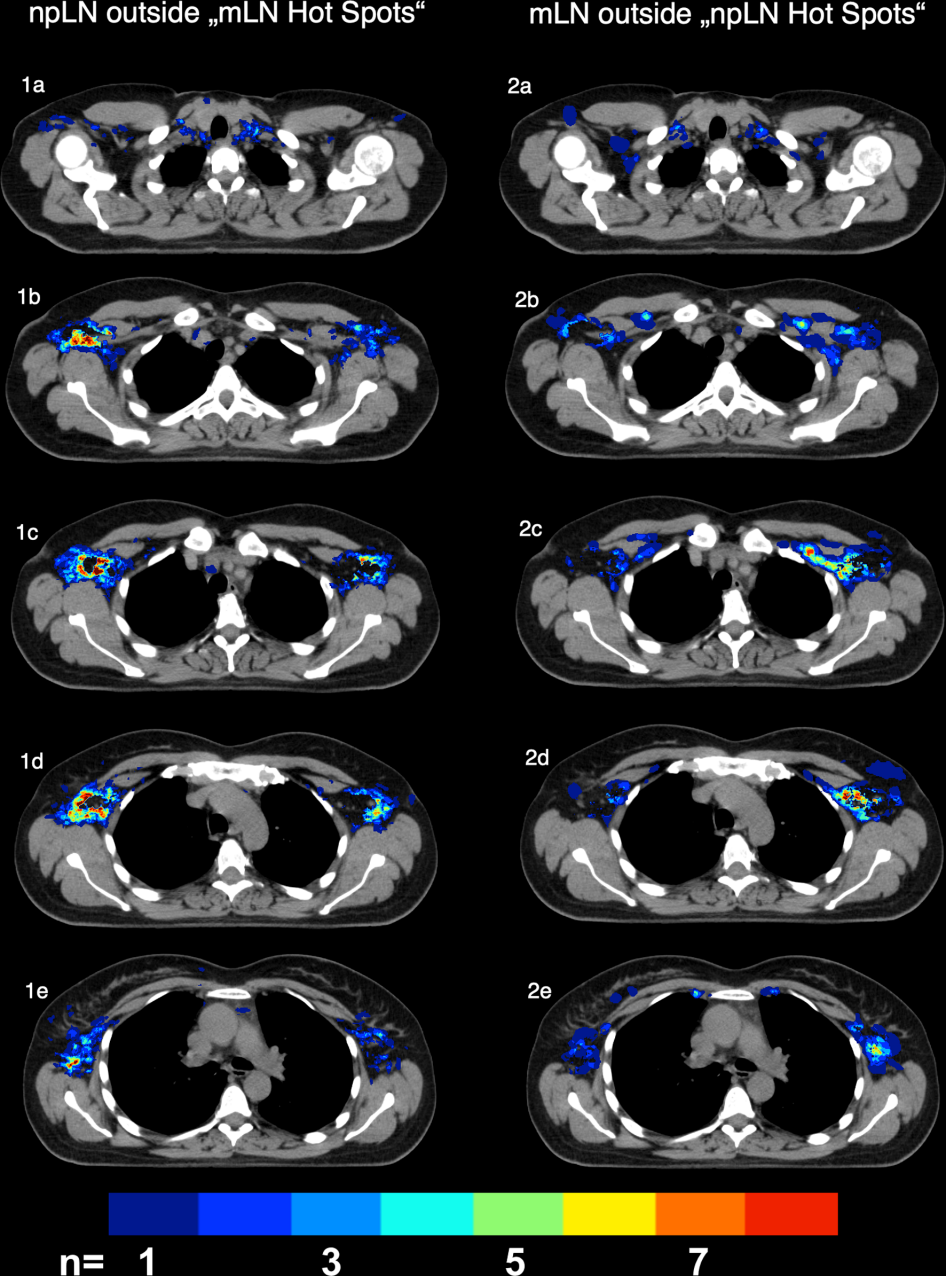
**1a–e** npLN outside mLN by blinding all areas with overlap of ≥ 3 mLN **2a–e** Delineation of lymph node metastases (mLN) outside areas with ≥ 3 mLN non-pathological lymph node metastases (npLN)

The comparison of mLN and npLN hot spots (Fig. 4) confirm that mLN accumulate more centrally, in close proximity to the subclavian vein. npLN on the other hand were observed more lateral, cranial and caudal of LMN hot spots.

The overlap of mLN and npLN differed with regard to the lymph node level. Table 2 summarizes the overlap between mLN and npLN and hotspots of npLN. The largest overlap between mLN and npLN can be found in level I. The smallest overlap between the LN were observed in Level III, the supraclavicular and the internal mammary region.

**Table 2:** Comparison of lymph node metastases (mLN) and non-pathologic lymph nodes (npLN) or npLN hot-spots, respectively. Number (n) and percent (%) of lymph nodes completely (> 95% volume), partly (5–95%) not (< 5%) overlapping npLN (or npLN hot spots) in the different lymph node levels

LN level	mLN n =	mLN versus npLN						mLN versus npLN “hot-spots”					
		Overlapping		Partly overlapping		Not overlapping		Overlapping		Partly overlapping		Not overlapping	
Level I	186	39	21.0%	133	71.5%	14	7.5%	5	2.7%	126	67.7%	55	29.6%
Level II	35	1	2.9%	30	85.7%	4	11.4%	0	0.0%	16	45.7%	19	54.3%
Level III	36	0	0.0%	21	58.3%	15	41.7%	0	0.0%	2	5.6%	34	94.4%
Level IV	31	1	3.2%	19	61.3%	11	35.5%	0	0.0%	5	16.1%	26	83.9%
IM	27	0	0.0%	6	22.2%	21	77.8%	0	0.0%	0	0.0%	27	100.0%
Other	11	0	0.0%	3	27.3%	8	72.7%	0	0.0%	0	0.0%	11	100.0%
Total	326	41	12.6%	212	65.0%	73	22.4%	5	1.5%	149	45.7%	172	52.8%

It is known, that recurrent mLN have a different distribution compared to primary mLN with more LN located in the “upper” lymph node levels (level III-IV). In our study mLN in recurrent and metastatic breast cancer exceeded more often the visible npLN. The relative overlapping volume was 39.2% for primary and 35.8% for recurrent cancer. For M0 the overlapping volume was 41.6% and for M1 33.7%.

## Discussion

In our study, remarkable differences between the distribution of mLN and npLN were found. Despite the large number of analyzed npLN, mLN occurred frequently outside areas with npLN appearance. In level II and III the overlap between mLN and npLN was particularly small.

Axillary lymph node dissection has been the standard procedure to assess the axillary lymph node status for many years. However, with establishment of SLNB and omission of ALND in a raising number of patients less information about axillary lymph node involvement is available. Since the extent of axillary lymph node involvement is crucial for indication of radiotherapy and systemic therapy, imaging of the axilla has gained importance. CT imaging is regularly used for staging purposes and treatment planning. However most earlier studies report a low sensitivity (46%) of CT imaging regarding the detection of lymph node metastases in breast cancer [16]. The low sensitivity can be attributed to the fact that distinction between mLN and physiological LN is based on size and shape of the lymph nodes only. Our 3D-comparison of mLN and npLN reveals that the location of a visible lymph node within the lymph node system can provide additional valuable information: For some regions (e.g. in Level III; Fig. 21c/2c). we observed a large discrepancy between high frequency of mLN and low frequency of npLN indicating a high pretest probability for mLN. Enlarged lymph nodes clearly visible in CT images in those areas are more likely to be pathological. Thus, they should be considered suspicious and require further diagnostic clarification.

Nodal irradiation aims to eradicate microscopic (invisible) tumor cells within the lymphatic drainage system. Thus, it is crucial to assess the extent of the drainage system and areas at risk. Previous studies have mapped mLN and compared them to recently published contouring atlases [17–19]. In an earlier analysis by our study group we observed that the majority of primary and recurrent mLNs are located within RTOG and ESTRO contouring margins [17–19]. Nevertheless, depending on the lymph node level, up 32% of mLN occured outside the recommended CTV margins. A crucial question that arises from our earlier work is whether the distribution pattern of the lymph node metastases simply results from the distribution of the physiological lymph nodes, or whether a certain metastatic pattern within the lymphatic system can be identified. This information is necessary to decide in clinical practice whether visible lymph nodes in the planning CT should always be included in the CTV.

MacDonald et al. [20] mapped benign and malign lymph nodes using nanoparticle-enhanced MRI and compared them to the RTOG contouring atlas used in radiotherapy. Even though benign and malign lymph nodes were delineated separately, no comprehensive comparison and analyses of the distribution was performed. In our current study, we compared mLN to contralateral “healthy” LN. This method provides the advantage of comparing mLN to the “normal” lymphatic drainage system without alterations caused by cancer or locoregional treatment. According to our results, areas that contain physiologically a large number of lymph nodes do not reliably correspond to the main locations for mLN. This indicates a large impact of other previously identified physiological (e.g. fluid pressure and microcirculation) and immunological factors (e.g. chemokine gradient) for lymphatic tumor spread [21, 22].

For the lateral parts of level I, a high frequency of visible lymph nodes in CT-images in the healthy axilla and a very low occurrence of lymph node metastases were observed. This leads to the assumption that visible lymph nodes in Level I outside the recommended contouring margins are likely to represent physiological LNs and don’t always need to be included in the CTV as long as the target volumes correspond to the recommend contouring guidelines and include mLN hot spots [15, 23].

For level II and III however, the number of visible lymph nodes in the healthy axilla was very low despite an accumulation of mLN in these areas. To account for this higher pretest-probability for mLN, inclusion of visible lymph nodes in the CTV of Level II and III should be considered in high risk patients (Fig. 32a–e).

A potential limitation of this study is that locoregional treatment to the axillary lymph nodes must be expected in a relevant part of the patient collective (including both primary and recurrent breast cancer). This potentially alters the pattern for lymph node metastases and may account for some of the differences observed between mLN and npLN. Nevertheless, locoregional treatment effect in particular level I and the lateral part of level II since they are regularly being included in surgical treatment and radiotherapy. The largest differences where however observed in level III and the medial part of level II.

## Conclusion

Distribution of mLN and npLN differ clearly within the lymph node system. Despite a large number of analyzed npLN, mLN occurred frequently outside areas with npLN appearance. In level II and III the overlap between mLN and npLN was particularly small. The results indicate that individual adaption of the recommended CTV-margins to include visible lymph nodes in CT-images in level II and III should be considered.

## Data Availability

The datasets used and/or analyzed during the current study are available from the corresponding author on reasonable request.

## Abbreviations

ALND: Axillary lymph node dissection
mLn: Lymph node metastases
npLN: Non-pathological lymph nodes
CT: Computed tomography

## References

[1] Early Breast Cancer Trialists’ Collaborative Group. Multi-agent chemotherapy for early breast cancer. Cochrane Database Syst Rev. 2002;(1):CD000487. 10.1002/14651858.CD000487. Update in: Cochrane Database Syst Rev. 2008;(4):CD000487. PMID: 11869577. 10.1002/14651858.CD000487 11869577

[2] Peto R, Davies C, Group EBCTC (2012). Comparisons between different polychemotherapy regimens for early breast cancer: meta-analyses of long-term outcome among 100,000 women in 123 randomised trials. Lancet.

[3] Network. NCC. Breast Cancer- Version 3.2020. https://www.nccn.org/professionals/physician_gls/pdf/breast.pdf. Accessed 13 Mar 2020.

[4] Whelan TJ, Olivotto IA, Parulekar WR (2015). Regional nodal irradiation in early-stage breast cancer. N Engl J Med.

[5] Poortmans PM, Collette S, Kirkove C (2015). Internal mammary and medial supraclavicular irradiation in breast cancer. N Engl J Med.

[6] Bromham N, Schmidt-Hansen M, Astin M, Hasler E, Reed MW (2017). Axillary treatment for operable primary breast cancer. Cochrane Database Syst Rev.

[7] Schaapveld M, Otter R, de Vries EG (2004). Variability in axillary lymph node dissection for breast cancer. J Surg Oncol.

[8] Henke G, Knauer M, Ribi K (2018). Tailored axillary surgery with or without axillary lymph node dissection followed by radiotherapy in patients with clinically node-positive breast cancer (TAXIS): study protocol for a multicenter, randomized phase-III trial. Trials.

[9] Natsiopoulos I, Intzes S, Liappis T (2019). Axillary lymph node tattooing and targeted axillary dissection in breast cancer patients who presented as cN+ before neoadjuvant chemotherapy and became cN0 after treatment. Clin Breast Cancer.

[10] Offersen BV, Boersma LJ, Kirkove C (2016). ESTRO consensus guideline on target volume delineation for elective radiation therapy of early stage breast cancer, version 1.1. Radiother Oncol.

[11] RTOG. Breast Cancer Atlas for Radiation Therapy Planning. https://www.rtog.org/LinkClick.aspx?fileticket=vzJFhPaBipE%3d&tabid=236. Accessed 21 June 2020.

[12] Antoch G, Saoudi N, Kuehl H (2004). Accuracy of whole-body dual-modality fluorine-18-2-fluoro-2-deoxy-D-glucose positron emission tomography and computed tomography (FDG-PET/CT) for tumor staging in solid tumors: comparison with CT and PET. J Clin Oncol.

[13] Riegger C, Koeninger A, Hartung V (2012). Comparison of the diagnostic value of FDG-PET/CT and axillary ultrasound for the detection of lymph node metastases in breast cancer patients. Acta Radiol.

[14] Ergul N, Kadioglu H, Yildiz S (2015). Assessment of multifocality and axillary nodal involvement in early-stage breast cancer patients using 18F-FDG PET/CT compared to contrast-enhanced and diffusion-weighted magnetic resonance imaging and sentinel node biopsy. Acta Radiol.

[15] Borm KJ, Voppichler J, Dusberg M (2018). FDG/PET-CT based lymph node atlas in breast cancer patients. Int J Radiat Oncol Biol Phys.

[16] Heusner TA, Kuemmel S, Hahn S (2009). Diagnostic value of full-dose FDG PET/CT for axillary lymph node staging in breast cancer patients. Eur J Nucl Med Mol Imaging.

[17] Chang JS, Byun HK, Kim JW (2017). Three-dimensional analysis of patterns of locoregional recurrence after treatment in breast cancer patients: validation of the ESTRO consensus guideline on target volume. Radiother Oncol.

[18] Chang JS, Lee J, Chun M (2018). Mapping patterns of locoregional recurrence following contemporary treatment with radiation therapy for breast cancer: a multi-institutional validation study of the ESTRO consensus guideline on clinical target volume. Radiother Oncol.

[19] Gentile MS, Usman AA, Neuschler EI, Sathiaseelan V, Hayes JP, Small W (2015). Contouring guidelines for the axillary lymph nodes for the delivery of radiation therapy in breast cancer: evaluation of the RTOG breast cancer atlas. Int J Radiat Oncol Biol Phys.

[20] MacDonald SM, Harisinghani MG, Katkar A, Napolitano B, Wolfgang J, Taghian AG (2010). Nanoparticle-enhanced MRI to evaluate radiation delivery to the regional lymphatics for patients with breast cancer. Int J Radiat Oncol Biol Phys.

[21] Nathanson SD (2003). Insights into the mechanisms of lymph node metastasis. Cancer.

[22] Jones D, Pereira ER, Padera TP (2018). Growth and immune evasion of lymph node metastasis. Front Oncol.

[23] Offersen BV, Boersma LJ, Kirkove C (2015). ESTRO consensus guideline on target volume delineation for elective radiation therapy of early stage breast cancer. Radiother Oncol.

